# Building Public Health Capacity through a Sustainable South–South–North Training Program

**DOI:** 10.4269/ajtmh.20-0273

**Published:** 2020-09-08

**Authors:** Taryn Clark, Manuela Verastegui, Freddy Tinajeros, Maritza Calderon, Rony Colanzi, Robert H. Gilman

**Affiliations:** 1 Department of Emergency Medicine, SUNY Downstate Medical Center, New York, New York;; 2 Kings County Hospital Center, New York, New York;; 3 Department of International Health, Johns Hopkins University Bloomberg School of Public Health, Baltimore, Maryland;; 4 A.B Prisma, Lima, Peru;; 5 Department of Cellular and Molecular Sciences, Universidad Peruana Cayetano Heredia, Lima, Peru;; 6 Infectious Diseases Research Laboratory, Universidad Peruana Cayetano Heredia, Lima, Peru;; 7 Hospital Universitario Japones, Santa Cruz de la Sierra, Plurinational State of Bolivia

## Abstract

Capacity building in public health is an urgent global priority. Recently, there has been an increasing emphasis on South–South and triangular cooperation. We describe our experience with a public health training collaboration between Peru and Bolivia, with Peru providing capacity building and expertise to Bolivia, while receiving supportive funding and training from the United States. This collaboration has led to a groundswell of research on clinically significant diseases, outreach to more than 800 scientists, several dozen publications, and the start of four institutional review boards. South–South and South–South–North collaborations should publish their experiences, and Northern funding organizations should consider funding such collaborations.

Public health capacity building in training, research, and policy is a global priority.^1^ In the last two decades, there has been an increasing emphasis on South–South collaboration (interaction between countries of the Global South) across all fields, but especially in medicine, where shared experience and resources can make a significant difference in meeting similar health challenges.^2^ Benefits of South–South cooperation include enhanced global health security, strengthened public health service delivery, and socioeconomic development, as well as a potential focus on social justice with emphasis on equity, shared values, and addressing social determinants of health.^3–5^

South–South and South–South–North (triangular cooperation, with the Northern country typically providing funding or technical expertise) collaborations have been recognized as “a model for global partnership for sustainable development,” and there are calls for more recognition of the value of South–South training from researchers in the Global South.^6^ There are multiple examples of South–South cooperation already within the field of medicine. Physician exchanges,^5,7^ disease-specific initiatives,^8,9^ and residency training programs^10^ have been discussed in the literature. However, South–South collaboration on research is lower than expected^2^ and, to the authors’ knowledge, there is no discussion of long-term, sustainable South–South or triangular research training programs for building public health capacity. This is unfortunate as lack of resources and research skills are a known barrier to South-led research.^11^

Over the last 25 years, a multi-institutional collaboration between Peruvian and Northern groups has developed into a remarkable training network within Peru, educating more than 95 Peruvian graduate and postgraduate students and more than 500 non-Peruvian students and publishing 500 original research articles in peer-reviewed journals. The principle Peruvian institution, Universidad Peruana Cayetano Heredia (UPCH), has been recognized as a leading research university, and Peru has become a research powerhouse in Latin America.^12,13^ Given the expertise that now exists in Peru, we began to use a South–South–North approach to strengthen the scientific research community in Bolivia. Through research and training grants, including a Fogarty D43 NIH training grant (making this an example of triangular cooperation), we have developed a sustainable public health capacity building program between Peru and Bolivia.

The Peruvian–Bolivian–United States South–South–North training program was formalized 5 years ago. Our group from Peru began to study congenital Chagas and Chagas cardiomyopathy, both of which are more prevalent and have much greater clinical significance in Bolivia than Peru. When we collaborated with Bolivian scientists, we realized that medical research in Bolivia was under-resourced. From here, we developed a plan to train Bolivian scientists in public health research. We initially did this through individual laboratory rotations in Peru specifically for our collaborators. After seeing the success of these specific investments, we eventually transitioned to the systematic program outlined in the following texts.

The program is built stepwise. Our first level of training is aimed at reaching as many scientists as possible. We hold multiple workshops and international conferences in Bolivia, with nearly 800 Bolivian participants to date, on research methodology, ethics, publishing, and specific diseases. From these broad introductions, we build a network of local scientists and physicians and select recruits for further training. Initially, trainees are brought from Bolivia to Peru for short rotations in basic science and research, lasting weeks to months. Some candidates then return to Peru for extended laboratory rotations or for masters programs in biostatistics and epidemiology at UPCH, which is closely modeled on the program at Johns Hopkins Bloomberg School of Public Health, and is directed by a graduate of our training program in Peru. Particularly dedicated trainees are assisted with applications to PhD programs in Latin America, North America, and Europe. Throughout the training steps, Bolivians have multiple opportunities to work on collaborative research projects with Peruvians focusing on diseases of import to Bolivia.

In addition to the formalized training program, we have a strong emphasis on multidisciplinary research, based on the values of ethics, collaboration, and generosity to improve the health and quality of life in our communities. In our experience, Bolivian trainees appreciate and learn from the exposure to cutting-edge laboratory and field science at UPCH and other Peruvian institutions both through the training program and through ongoing research projects. The introduction to high-end research in another Latin American country has enabled trainees to understand what could be accomplished in Bolivia, leading to a groundswell of research on antibiotic resistance, Chagas disease, tuberculosis microbiology, neurologic complications in HIV, and Zika, all of which are diseases with clinical significance in Bolivia.

More than a dozen Bolivian trainees have gone on to attend courses in the United States, approximately half a dozen have attended advanced laboratory rotations at Johns Hopkins School of Public Health, and four have presented research at international meetings. Trainees have gone on to a clinical fellowship at Imperial College, a PhD program at Cambridge, a PhD program at Johns Hopkins School of Public Health, and one trainee will start a PhD program at the University of North Carolina this fall. Several more trainees are currently applying for advanced training programs in Peru, the United States, and Europe. Through the actions of the South–South–North collaboration, four institutional review boards (IRBs) have been developed in Bolivia, including the first IRB in the city of Santa Cruz, Bolivia’s most populous city.^14^ Thirty-five articles have been published, and at least a dozen more are in various stages of preparation, most spearheaded by Bolivian trainees from the formal training program. One of our Bolivian mentors has noted that these articles have great curricular value for the participating authors, and publication of these articles promotes further research both by local scientists and in local institutions.

Historically, over 90% of Peruvian researchers who trained with this program still work primarily in Peru and other countries in Latin America, thus contributing back to their community and limiting brain drain.^14^ Based on this experience, we expect that most Bolivians who train with our program will return to their home communities to strengthen local public health research networks. In our limited experience over the last 5 years, this has held true.

Although significant funding currently comes from the Global North, there has been a recent shift to more locally funded collaborations. For example, Hospital de la Mujer Dr. Percy Boland built a quantitative polymerase chain reaction (qPCR) laboratory for collaborative research, and Universidad Católica Boliviana San Pablo built a tuberculosis research laboratory. The qPCR laboratory at Hospital de la Mujer is used for research on congenital Chagas, toxoplasmosis, and human papillomavirus and is currently being used to diagnose COVID-19 as the regional laboratories are overwhelmed. The tuberculosis laboratory at Universidad Católica performs microscopic observation drug susceptibility testing in addition to ongoing research on tuberculosis in Bolivia.^14^ Training for both laboratories was carried out initially in Peru, then in Bolivia with Peruvian supervision, and now is primarily run by Bolivian scientists.

Through the last 5 years, we have learned several important lessons. First, there is great value in emphasizing cooperation and teamwork. Collaboration among multiple laboratories, universities, hospitals, institutions, and the Ministry of Health allows for synergy and improved research outcomes. Second, moving systematically through training steps allows us to educate the highest number of people and helps select the most appropriate candidates for more in-depth training. Third, allowing Bolivian scientists to determine issues of import to their communities helps us to focus on clinically significant diseases and gives ownership of the projects to Bolivian scientists.

We have several goals for the future. We would like to see further investment and funding from the Bolivian government and nongovernmental organizations. Several of our program members are actively working with the Bolivian government on this goal. We hope to train at least five more PhD-level scientists and 10 more masters-level scientists and recently received another 5 years of funding from the Fogarty group to help achieve this goal. We are working to fully transition our associated research laboratories over to Bolivian scientists who can both head projects and train new scientists. We would like to build stronger ties between Peruvian and Bolivian institutions to increase cooperation and share research work. Finally, we would like to see Bolivia continue to develop its own medical research capacity. Several of our trainees have started projects with the support of the Bolivian government, including in the area of COVID-19 treatment. We support the expansions of such projects.

Given the success we have seen with this South–South capacity building program between Peru and Bolivia, we would like to see further development of comparable programs. Cooperation between countries with similar cultural histories, languages, and socioeconomic disparities allows for effective mutual development. Further research, publications, and quantitative results on South–South and triangular cooperation in public health may help induce funders from the Global North to support future collaborations. There are many well-established research groups in the Global South that could formalize and publicize their South–South efforts, as well as expand their reach. Publicizing and replicating these efforts will be key to establishing strong public health networks in the Global South.
